# A Randomized, Double‐Blinded Pilot Study Comparing Synthetic Versus Human‐Derived Topical Epidermal Growth Factor for Facial Rejuvenation and Psychosocial Perception

**DOI:** 10.1111/jocd.70927

**Published:** 2026-05-18

**Authors:** Brittany Abud, Lander McGinn, Elvira Cawthon, Ashley Pankey, Nimit Gandhi, Catherine Carvajal, Steven Dayan

**Affiliations:** ^1^ Department of Otolaryngology ‐ Head and Neck Surgery University of Illinois College of Medicine Chicago Illinois USA; ^2^ Cawthon Clinical Consulting Hendersonville Tennessee USA; ^3^ DeNova Research Chicago Illinois USA; ^4^ Division of Facial Plastic and Reconstructive Surgery, Department of Otolaryngology University of Illinois at Chicago Chicago Illinois USA

**Keywords:** aging face, epidermal growth factor, facial rejuvenation, non‐invasive, skincare, topical

## Abstract

**Background:**

Topical growth factor‐containing cosmeceuticals are widely used to improve visible signs of facial photoaging. Interest has increased in peptide‐based alternatives that aim to support similar regenerative pathways without relying on human‐derived biologics.

**Aim:**

To compare the clinical efficacy, tolerability, and exploratory psychosocial perception outcomes of a topical recombinant epidermal growth factor (REGF), oligopeptide‐1, serum with a human‐derived growth factor (operationally labeled HDEGF) serum.

**Methods:**

This was a 12‐week, prospective, randomized, double‐blinded, parallel‐group pilot trial involving 20 adult female participants (50–65 years), predominantly non‐Hispanic White with Fitzpatrick Skin Types II and III with moderate to severe facial photoaging. Participants applied their assigned serum twice daily and used a standardized cleanser and sunscreen regimen. Primary endpoints included investigator‐rated changes in skin texture and firmness. Secondary endpoints included participant‐reported satisfaction. Tertiary endpoints evaluated psychosocial perception using standardized photographs rated by blinded lay evaluators.

**Results:**

Investigator ratings favored the REGF cohort for improvements in skin texture and firmness at later study visits, while global wrinkle severity ratings changed minimally in both cohorts. Participants in the REGF cohort reported higher satisfaction across multiple questionnaire items. In exploratory lay‐evaluator assessments, small between‐visit differences were observed in select perception domains, including estimated age.

**Conclusion:**

In this pilot study, the REGF formulation was associated with greater improvement in selected investigator‐ and participant‐reported outcomes compared with the HDEGF formulation over 12 weeks. Perception‐based findings are exploratory and limited by the non‐validated nature of the rating instrument. Interpretation is further limited by the small, female‐only sample and short follow‐up; larger, longer, and more diverse studies are warranted.

## 1 Introduction

Skin aging is a multifactorial process characterized by the progressive deterioration of skin structure and function due to intrinsic (chronological aging) and extrinsic (environmental) factors [1]. Photoaging is predominantly caused by harmful ultraviolet (UV) radiation, which inflicts significant damage to the skin [2]. Other hallmarks of aging skin include the appearance of fine lines, wrinkles, loss of elasticity, uneven skin tone, and reduced hydration. The demand for topical anti‐aging treatments has surged as consumers seek effective alternatives to invasive procedures. Biologically active ingredients used as a topical product have been shown to be effective in improving facial aesthetic appearance, including rhytids [3].

Beyond structural deterioration, aging skin reflects progressive dysregulation of intercellular signaling networks that coordinate repair, matrix homeostasis, and barrier function. Topical formulations containing human fibroblast conditioned media (hCCM) have been used in skincare since the early 2000s and are often described as a fibroblast‐derived secretome encompassing growth factors, cytokines, peptides, enzymes, and extracellular vesicles [4, 5].

Among the most established formulas is TNS Advanced+ Serum (SkinMedica), which incorporates human fibroblast conditioned media (hCCM) derived from neonatal dermal fibroblast cultures, along with supportive peptides—intended to augment regenerative signaling relevant to epidermal renewal and dermal matrix homeostasis [4]. Preliminary consumer panel findings have demonstrated its efficacy in reducing visible signs of aging, making it a widely used product in dermatology and aesthetic medicine [1, 6, 7].

However, interest has grown in alternatives that avoid human‐derived growth factors, which may pose ethical considerations for some patients and clinicians [8, 9]. Publications by both medical professionals in the aesthetics industry as well as market research have demonstrated this mounting consumer demand [10, 11]. One such product, Needle‐less Growth Factor by DRMTLGY, purports to offer comparable clinical benefit—without relying on human biologics. Instead, it employs synthetic peptides, namely oligopeptide‐1, otherwise known as recombinant epidermal growth factor (REGF).

This peptide has been well‐studied for its ability to promote wound healing, modulate inflammatory pathways, and stimulate keratinocyte and extracellular matrix proliferation [12, 13, 14]. Targeted human studies have also demonstrated the efficacy of this peptide on improving the texture of photoaged skin [15]. The anti‐aging effects of both products leverage biological pathways analogous to wound healing, a mechanism widely supported in peer‐reviewed literature [13, 16, 17, 18].

Furthermore, while previous studies have emphasized objective skin improvement, relatively few investigations have assessed how such changes might influence psychosocial perception, such as perceived age or broader social impression, which increasingly influence patient‐reported satisfaction. Appearance‐based cues can shape impression formation, including judgments related to vitality, competence, and social appeal [19]. This phenomenon has been investigated most extensively within surgical and minimally invasive aesthetic domains to improve facial harmony and social perception [20, 21]. The present study builds upon prior work to explore whether non‐surgical, topical interventions may also produce measurable changes in appearance‐related perception.

Despite the broad adoption of growth factor–containing cosmeceuticals for facial rejuvenation, the clinical literature remains heterogeneous. In a 2023 systematic review (2000–October 2022), 33 studies (9 randomized controlled trials) representing 1 180 participants evaluated 23 topical growth factor preparations, typically applied twice daily for a mean duration of three months. Investigator‐assessed outcomes suggested modest median improvements in skin texture (< 50%), fine lines/wrinkles (< 35%), and overall facial appearance (< 20%) vs. baseline, while participant‐reported improvement was generally higher; comparative RCTs did not consistently demonstrate superiority of one active regimen over another [22]. These limitations—alongside variability in growth factor sourcing, co‐ingredients, and outcome standardization—support the need for controlled, product‐specific comparisons using reproducible endpoints.

This pilot randomized, double‐blinded clinical trial was designed to compare the efficacy and tolerability of a serum employing REGF vs. one employing HDEGF over a 12‐week period. In the present manuscript, we use HDEGF as an operational label for the study arm receiving the human‐derived conditioned‐media formulation (TNS Advanced+ Serum), to distinguish it from the REGF arm receiving oligopeptide‐1‐based peptide technology. Primary endpoints included investigator‐ and subject‐rated improvements in facial skin quality, while secondary and tertiary measures aimed to evaluate patient satisfaction and projected first impressions, respectively. In so doing, this study seeks to provide an early‐stage, evidence‐informed framework to assist aesthetic clinicians in selecting topical agents aligned with both clinical goals and patient preferences.

## 2 Methods

### 2.1 Study Design

This pilot investigation was conducted as a 12‐week, prospective, randomized, double‐blinded, parallel‐group clinical trial with a primary objective to explore and compare the effects of two topical formulations employing epidermal growth factor, either REGF or HDEGF, on facial skin rejuvenation. The study secondarily aimed to assess participants' self‐perceived facial rejuvenation improvement after use of each serum. As a tertiary objective, the study investigated the psychosocial impact of each topical treatment arm. Participants were not compensated for participation.

All participants were first assigned a skincare regimen beginning with a gentle cleanser to be used twice daily. Subjects then applied their assigned product to the full face twice daily for 12 weeks. Participants were instructed to maintain stable lifestyle factors for the duration of the study, with no changes to sleep patterns, diet, or activity levels. Lastly, all subjects in both cohorts applied a broad‐spectrum sunscreen once daily, which was supplied by the same manufacturer as the product in the REGF cohort. These adjunctive products were standardized to ensure consistency across treatment arms and minimize confounding variables related to basic skin care. Residual confounding due to product synergy bias of adjunct product selection cannot be fully excluded, though this is addressed later as a limitation.

Participants were assessed at a baseline visit (Visit 1) and then again at 1, 2, and 3 months (Visits 2, 3, and 4). At each visit, blinded investigators assessed objective outcomes including an adapted version of the Wrinkle Severity Rating Scale (WSRS) for assessment of full facial wrinkles, and the Global Aesthetic Improvement Scale (GAIS), which is a validated global cosmetic outcomes assessment tool [23, 24]. Investigators also completed a Percentage Improvement Scale assessing perceived improvement in rhytids, skin texture, and skin firmness at each visit relative to baseline on a quartile‐based ordinal scale (0%–25%, 26%–50%, 51%–75%, or 76%–100% improvement). This Percentage Improvement Scale has not been validated and was used as an exploratory investigator‐rated endpoint. Finally, VISIA‐CR 5 imaging was captured by trained study personnel using the manufacturer's standardized protocol in a controlled setting, obtaining frontal and bilateral oblique views at each visit. VISIA‐derived outputs were normalized on a 0–1 scale, with lower scores indicating lesser visibility of the skin feature, reflecting more favorable outcomes.

Participants then completed subjective evaluations of their results using the same quartile‐based Percent Improvement Scale as investigators and a 22‐item Subject Satisfaction Questionnaire (Appendix S1) administered at weeks 8 and 12.

Finally, fifty independent, blinded lay evaluators rated standardized 2D frontal photographs of participants from baseline and final visits. Evaluators were blinded to treatment group and visit timing. Evaluators rated multiple impression‐based domains (e.g., dating success, occupational success, attractiveness, financial success, and athletic success) on a scale of 1 (strongly disagree) to 10 (strongly agree) and estimated each subject's age. This instrument was developed for exploratory purposes and is not a validated measure of these constructs.

Participants were randomized in a 1:1 ratio to receive a commercially available serum containing REGF or human‐derived conditioned‐media serum (operationally labeled HDEGF). Allocation was carried out via a computerized random number generator to minimize allocation bias. Both participants and investigators were blinded to the assigned treatment throughout the study duration. The study protocol was approved by the institutional review board (WCG IRB, in Cary, NC, USA, protocol #20250256) and adhered to accepted standards for Good Clinical Practice. All subjects provided written informed consent prior to study enrollment.

### 2.2 Subjects

Eligible patients included females aged 30–65 years with moderate to severe facial photoaging (scored 6–9 on the 10‐point modified Griffith's scale). Inclusion criteria further required good general health (ASA class I), any Fitzpatrick skin classification, and willingness to abstain from other facial treatments, such as injectables, surgery, and other topical and oral skin care regimens, for the duration of the study, with a preceding two‐week washout period. Exclusion criteria included facial procedures (e.g., injectable neuromodulator, hyaluronic acid filler, resurfacing, or surgical procedure) within the past 3 months, active dermatologic conditions (e.g., vitiligo, psoriasis, rosacea), use of prescription medication for dermatologic conditions, abnormal photosensitivity, use of tanning beds or self‐tanner within the past 3 weeks, or pregnancy.

Twenty female participants, aged 50 to 65 years, meeting the inclusion criteria were enrolled. Potential participants were identified through review of the electronic health record patient database at the study's center, during clinic visits, and via physician referrals. Interested individuals were approached either directly during a clinic visit or by telephone after record review. They then underwent pre‐screening to assess eligibility based on age, medical history, and study‐specific criteria, which were conducted by trained study personnel via telephone, electronic communication, or in person. Eligible individuals attended a screening visit where written informed consent was obtained prior to any study procedures, followed by confirmation of eligibility through medical history review and concomitant medication assessments. Participants who met all eligibility criteria were enrolled, with selection based solely on study requirements without regard to race, ethnicity, socioeconomic status, or other non‐medical factors.

### 2.3 Safety Monitoring

Participants were monitored at each visit for adverse events, including allergic reactions and signs of intolerance. There were no serious adverse events to report. No invasive procedures were performed, and all products used in this study are commercially available.

### 2.4 Statistical Analysis

Statistical analysis was performed using MedCalc Statistical Software version 23.5.1 (MedCalc Software Ltd., Ostend, Belgium; https://www.medcalc.org; 2026). Descriptive statistics were computed for all outcome measures across study visits. Demographic variables were compared between the two groups using two‐tailed independent samples *t*‐tests for continuous variables and either Fisher's Exact Test or two‐sample tests of proportion for categorical variables, as appropriate.

Significance testing of between‐group differences across assessments for non‐parametric ranked order data, such as investigator ratings on the Global Aesthetic Improvement Scale (GAIS), was performed using the Mann–Whitney *U* test (Wilcoxon rank‐sum test). The standardized effect size was derived from the Mann–Whitney *U* test, calculated as $r=|Z|/\sqrt{N}$, where Z is the standardized test statistic, and N is the total sample size. The absolute value of r is classified as small (≥ 0.1), medium (≥ 0.3), or large (≥ 0.5), following Cohen's thresholds. Changes in ordinal rating categories over time (e.g., improvement, no change, or worsening) were evaluated using Fisher's Exact Test to determine the significance of directional shifts.

VISIA‐CR 5 imaging data were analyzed both within and between groups. A series of one‐way analysis of variance (ANOVA) was used to evaluate changes across time points within each treatment group. The assumption of normality of residuals was verified using the Shapiro–Wilk test for normality, with *p* > 0.05 supporting normal distribution of the residuals per population. The assumption of homoscedasticity (homogeneity of variance) across samples was verified using Levene's Test, with *p* > 0.05 indicating homogeneity of variances across samples.

To compare differences in mean change from baseline between groups, two‐tailed independent samples *t*‐tests were applied to each facial feature analyzed. The assumption of normality (normal distribution of the samples) for each population was verified through application of the Shapiro–Wilk Test (*p* > 0.05), and the assumption of homogeneity of variance was verified using Levene's Test of Equality of Variances (*p* > 0.05). Where applicable, standardized effect sizes were calculated and reported to characterize the magnitude of observed effects.

To correct for the increased risk of type I error and potential false positive significance determinations due to the multiple outcomes, variables, timepoints, and therefore statistical comparisons performed, False Discovery Rate (FDR) Control using the Benjamini‐Hochberg procedure was applied, using an FDR of 0.05 (5%). Only *p*‐values remaining significant after application of FDR are reported.

## 3 Results

All twenty enrolled participants completed the study per protocol. Recruited participants were female, aged 50–65 years, predominantly non‐Hispanic White with Fitzpatrick Skin Types II and III. There were no statistically significant differences in age, ethnicity, race, or skin type distribution between the two treatment groups (all *p* > 0.05). Participant demographics are presented in Table 1.

**TABLE 1:** Participant demographics. enrolled subjects across both formulation groups were female, aged in the mid to upper 50's, predominantly non‐Hispanic white (caucasian) with fitzpatrick skin types II and III. There were no statistically significant differences identified in any demographics assessed between the REGF and HDEGF formulation groups.

	REGF group	HDEGF group	Statistical outcome
Gender	10 females	10 females	n/a
Age (mean, years)	59.4	56.3	Two‐tailed *t*‐test: t = −1.46, *p* = 0.162
Race	3 Hispanic 7 Non‐Hispanic	0 Hispanic 10 Non‐Hispanic	Fisher's Exact Test *p* = 0.2105
Ethnicity	9 White 1 East Asian	8 White 1 South East Asian 1 African American	Two‐sample proportions test z = 0.626, *p* = 0.531
Fitzpatrick skin type	5 Type II 3 Type III 2 Type V	2 Type II 5 Type III 3 Type IV	Fisher's Exact Test *p* = 0.112

### 3.1 Primary Outcomes: Investigator‐Assessed Efficacy

Across the 12‐week treatment period, neither product recorded a change in ratings on the adapted version of the WSRS across assessments (Table 2).

**TABLE 2:** Facial wrinkles scale. summary descriptives for the REGF and HDEGF formulation groups at visit 1 and visit 4 demonstrated a lack of change across assessments by blinded evaluators for both groups. Ratings on the Facial Wrinkles Scale, an adaptation of the WSRS, ranged from 1: Absent wrinkling; 2: Shallow but visible wrinkling; 3: Moderately deep wrinkling; 4: Deep wrinkling with well‐defined edges; and 5: Very deep wrinkling with redundant folds.

	REGF group		HDEGF group	
	Visit 1	Visit 4	Visit 1	Visit 4
Mean	3.8	3.8	3.7	3.7
SD	0.79	0.79	0.67	0.67
Median	4	4	4	4
Range (min, max)	3, 5	3, 5	3, 5	3, 5

**FIGURE 1:**
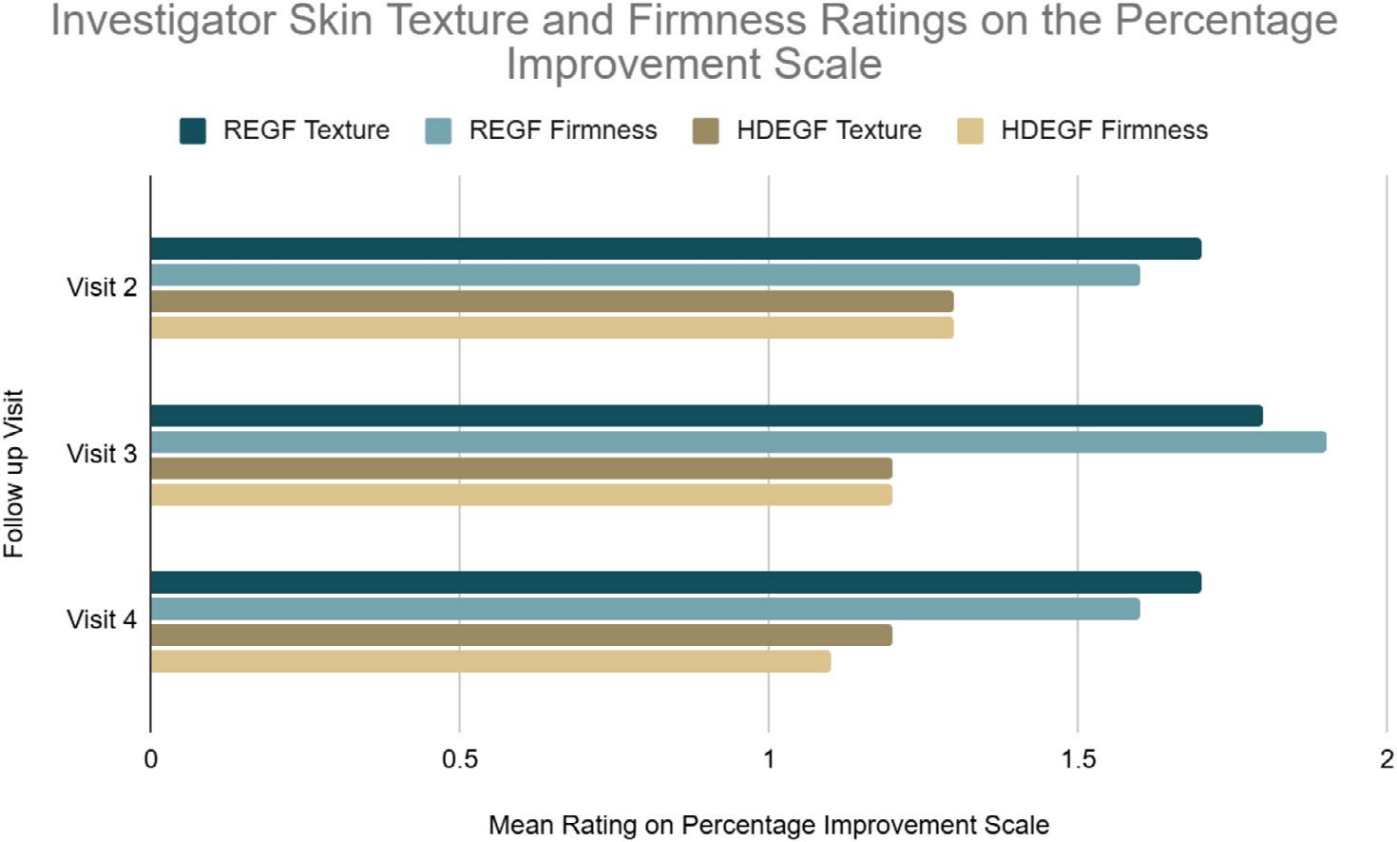
Investigator‐rated Percentage Improvement Scale (quartile‐based, anchored to baseline) for skin texture and firmness by visit and treatment cohort. A 1‐point change corresponds to a 25% increment in perceived improvement.

The Percentage Improvement Scale trended in favor of REGF across treatment visits. At Week 8 (Visit 3) assessment, investigators rated skin firmness on the Percentage Improvement Scale as having improved 26%–50% relative to baseline for 90% of subjects in the REGF group compared with 20% of subjects in the HDEGF group, a statistically significant difference (*p* = 0.0017). Similarly, for skin texture, ratings on the Percentage Improvement Scale at Week 8 relative to Baseline were rated by investigators as having improved 26%–50% for 80% of subjects in the REGF group compared with 20% of subjects in the HDEGF group, a statistically significant difference (*p* = 0.0073) (Figure 1). At Week 12, investigators rated 26%–50% improvement in skin texture in 70% of the REGF group and 20% of the HDEGF group, and 26%–50% improvement in skin firmness in 60% and 10% of the groups, respectively; these between‐group differences were not statistically significant. (Table 3).

**TABLE 3:** Investigator perceived improvement scale. investigator‐perceived percentage improvements for skin texture and firmness at visits 3 and 4, relative to baseline, for the REGF and HDEGF formulation groups, showing higher investigator‐rated percentage improvement for subjects in the REGF group compared with the HDEGF group at both assessments. Ratings on the Investigator Perceived Improvement Scale ranged from 1: 0%–25%; 2: 26%–50%; 3: 51%–75%; 4: 76%–100%.

Skin texture	Visit 3	Visit 4
**REGF group**		
1: 0%–25%	2 (20%)	3 (30%)
2: 26%–50%	8 (80%)	7 (70%)
3: 51%–75%	—	—
4: 76%–100%	—	—
**HDEGF Group**		
1: 0%–25%	8 (80%)	8 (80%)
2: 26%–50%	2 (20%)	2 (20%)
3: 51%–75%	—	—
4: 76%–100%	—	—

Skin firmness	Visit 3	Visit 4
**REGF Group**		
1: 0%–25%	1 (10%)	4 (40%)
2: 26%–50%	9 (90%)	6 (60%)
3: 51%–75%	—	—
4: 76%–100%	—	—
**HDEGF Group**		
1: 0%–25%	8 (80%)	9 (90%)
2: 26%–50%	2 (20%)	1 (10%)
3: 51%–75%	—	—
4: 76%–100%	—	—

**FIGURE 2:**
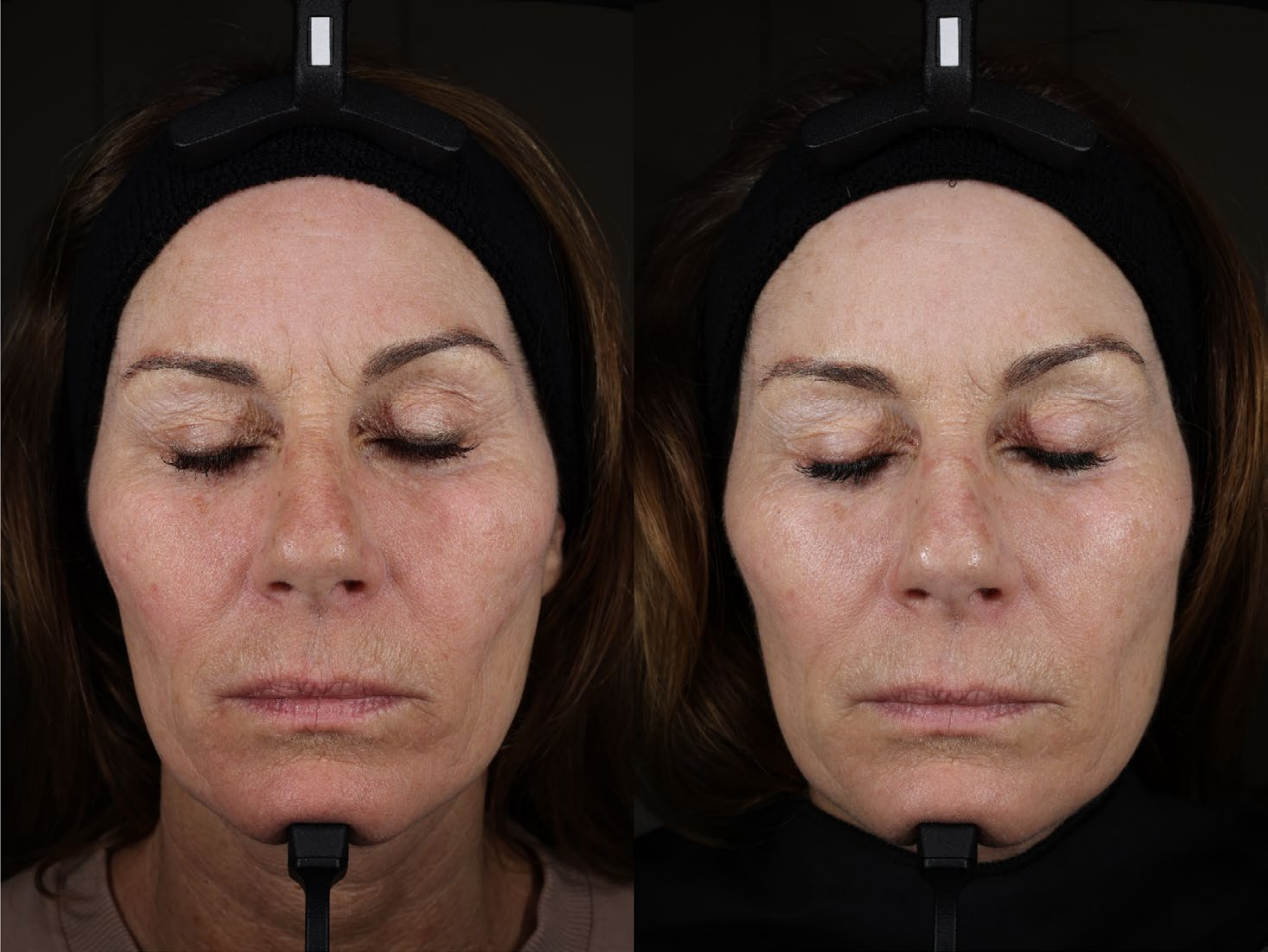
Before (left) and after (right) images of one subject within the REGF cohort, representing improvements in GAIS and UV spots detected on VISIA‐CR5 imaging.

**FIGURE 3:**
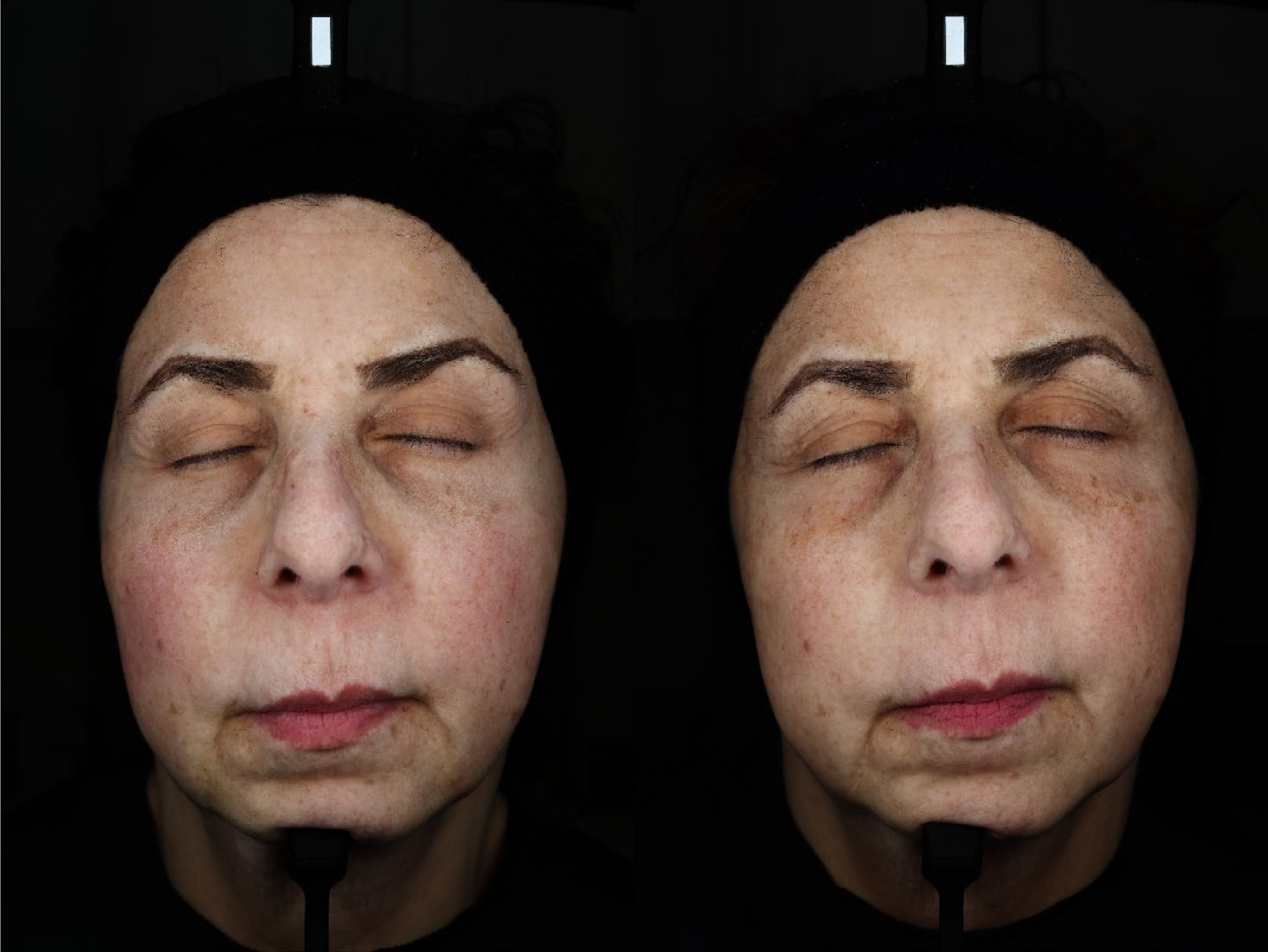
Before (left) and after (right) images of one subject within the HDEGF cohort, representing improvements in red features detected on VISIA‐CR5 imaging.

**FIGURE 4:**
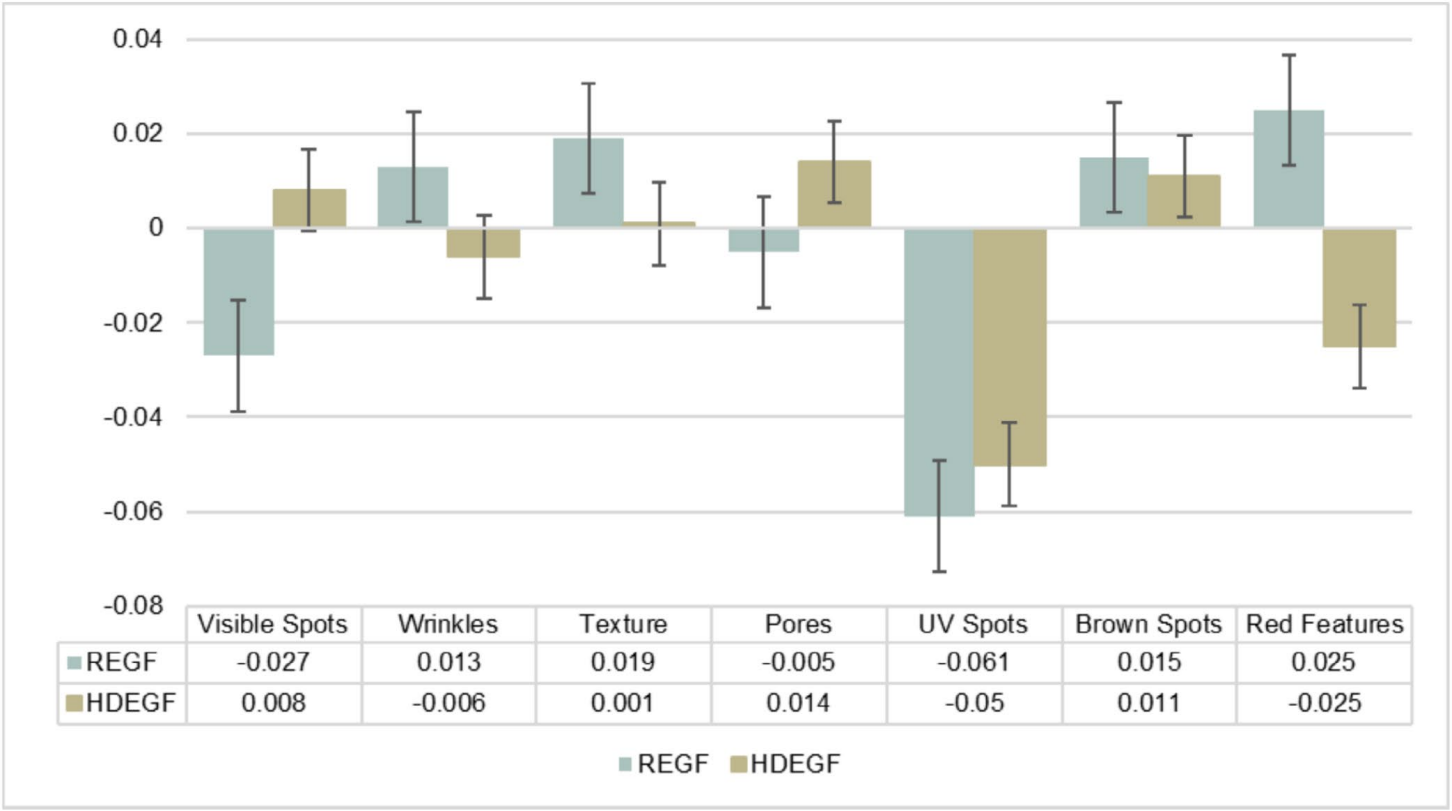
Investigator‐rated VISIA‐CR feature severity scores recorded at Week 12 (Visit 4) relative to Baseline for both formulation groups for each of visible spots, wrinkles, texture, pores, UV spots, brown spots, and red features. Scores were normalized between 0 and 1 such that lower scores indicated lesser detection of the assessed feature.

With regards to the Investigator GAIS (I‐GAIS) assessments at Week 12 (Visit 4) relative to Baseline, 90% of participants within the REGF group were rated as “Improved” compared to 60% in the HDEGF group (Table 4). This difference, however, was not found to be statistically significant (*p* > 0.05). Representative outcomes from subjects within each cohort are depicted in Figure 2 and Figure 3. VISIA‐derived measures showed variable directional changes across features in both groups. No statistically significant between‐group differences were identified for the VISIA endpoints at Week 12 (all *p* > 0.05) (Figure 4).

**TABLE 4:** Investigator global assessment of improvement scale (I‐GAIS). investigator GAIS ratings at visit 4, as considered relative to Baseline, showed a higher proportion of ‘Improved’ ratings in the REGF group at Visit 4. Ratings on the I‐GAIS ranged from 1: Worse; 2: No Change; 3: Improved; 4: Much Improved; to 5: Very Much Improved.

	REGF group	HDEGF group
5: Very Much Improved	—	—
4: Much Improved	—	—
3: Improved	9 (90%)	6 (60%)
2: No Change	1 (10%)	4 (40%)
1: Worse	—	—

### 3.2 Secondary Outcomes: Participant Self‐Assessments

The proportion of ‘Yes’ responses for subjects in the REGF group was greater for 16 of the 22 (73%) items in the questionnaire compared with the HDEGF formulation group at Visit 3, and 13 of the 22 (59%) items at Visit 4, indicating greater overall satisfaction with the outcome of the formulation use for subjects who used the REGF formulation compared with subjects who used the HDEGF formulation. At the 12‐week follow‐up, participants continued to report greater efficacy within the REGF treatment arm. Most notably within the questionnaire, 100% of participants in the REGF arm reported the product made their skin feel smoother both at weeks 8 and 12, compared to 70% and 80% respectively for those within the HDEGF treatment arm (*p* > 0.05). No item within the questionnaire received unanimous approval from those within the HDEGF treatment group.

**FIGURE 5:**
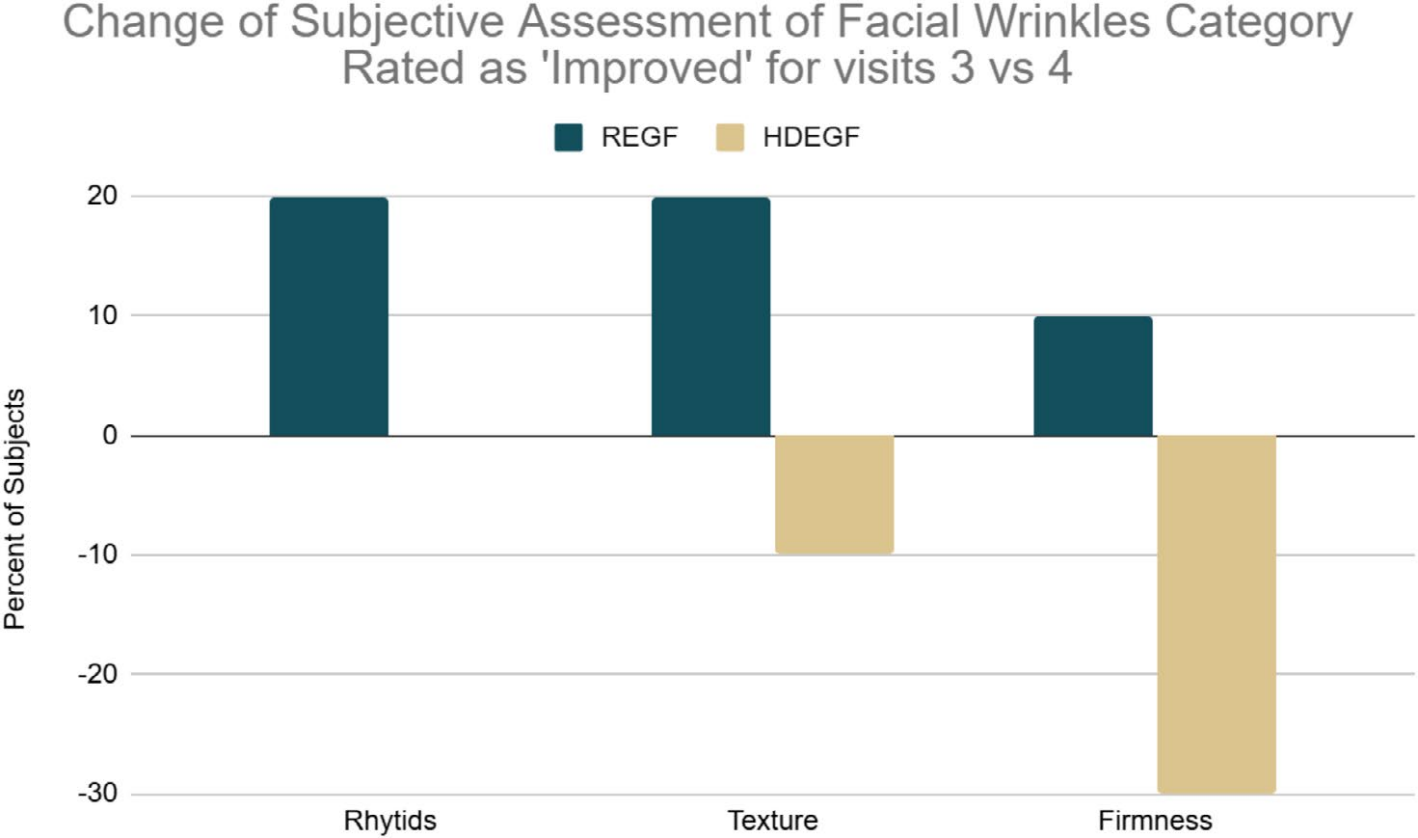
Participant‐rated Percentage Improvement Scale across Visits 3 and 4 (study completion) for perceived rhytids, skin texture, and skin firmness. A greater proportion of participants in the REGF cohort continued to report improvement over time compared with the HDEGF cohort.

Across all domains assessed in the participant‐rated Percentage Improvement Scale, most participants reported some degree of improvement over baseline in both formulations. With regard to skin texture and skin firmness, participants in the REGF cohort reported greater improvement compared with participants in the HDEGF cohort. When comparing changes over time, a greater proportion of participants in the REGF cohort continued to report improvement across the study (Figure 5).

### 3.3 Tertiary Outcomes: Psychosocial Impact

Lay evaluators perceived subjects within the REGF group as having improved Dating Success and Attractiveness. These changes, however, were not statistically significant (*p* > 0.05). However, a statistically significant increase in Athletic Success (*p* < 0.0001) between baseline and final visits was identified. However, this finding had a small associated effect size (*r* = 0.09).

Lay evaluator ratings showed mixed directional changes across domains, with individual domains for each group represented in Table 5.

**TABLE 5:** Lay evaluators' first impressions. parameters perceived as improved are bolded. Changes that are statistically significant, either positive or negative, are also bolded.

First impression category	Visit 1 to 4 change	*t*‐value	*p*	SIgnificance
**REGF**				
Social Skills	−0.48	−6.06	< 0.0001	***p* < 0.0001**
Academic Performance	−0.22	−2.64	0.0086	***p* < 0.01**
Dating Success	**+0.09**	+1.23	0.219	*p* > 0.05
Occupational Success	−0.14	−1.52	0.129	*p* > 0.05
Attractiveness	**+0.11**	+1.35	0.178	*p* > 0.05
Financial Success	−0.14	−1.62	0.106	*p* > 0.05
Relationship Success	−0.16	−2.06	0.040	***p* < 0.05**
Athletic Success	**+0.49**	+5.74	< 0.0001	***p* < 0.0001**
**HDEGF**				
Social Skills	−1.03	−10.46	< 0.0001	***p* < 0.0001**
Academic Performance	−0.98	−8.86	< 0.0001	***p* < 0.0001**
Dating Success	−0.37	−3.69	0.0025	***p* < 0.005**
Occupational Success	−0.79	−7.32	< 0.0001	***p* < 0.0001**
Attractiveness	−0.38	−3.67	0.00027	***p* < 0.0005**
Financial Success	−0.75	−7.04	< 0.0001	***p* < 0.0001**
Relationship Success	−0.74	−7.64	< 0.0001	***p* < 0.0001**
Athletic Success	**+0.10**	+1.06	0.290	*p* > 0.05

Finally, evaluator assessment of subject age at each of Visit 1 and Visit 4 resulted in both cohorts being perceived as younger at Visit 4, by a mean of 1.47 years (*p* < 0.0001) for subjects in the REGF cohort and a mean of 1.0 years (*p* < 0.005) for subjects in the HDEGF group.

## 4 Discussion

This pilot randomized, double‐blinded trial compared a topical peptide‐based epidermal growth factor serum (REGF) with a human‐derived conditioned‐media serum (HDEGF) over 12 weeks. Investigator‐ and participant‐rated outcomes suggested greater improvement in skin texture and firmness in the REGF cohort, whereas the adapted WSRS and most VISIA‐derived measures showed limited separation between groups. These findings are broadly consistent with the topical growth factor literature, in which improvements are often reported for selected skin quality parameters over approximately 3 months, although direct comparative evidence remains limited and heterogeneous [22, 25, 26].

Prior studies have reported favorable changes in photoaged skin with both recombinant EGF (oligopeptide)‐based formulations and fibroblast‐conditioned media products [4]. The present study expands upon this literature by providing preliminary head‐to‐head data. However, clinical performance of topical biologically active products depends not only on the nominal active ingredient but also on vehicle composition, concentration, stability, and cutaneous bioavailability [22, 27, 28]. Accordingly, these data do not establish superiority of peptide‐based technology over conditioned‐media formulations; rather, they suggest that the specific REGF formulation under investigation may produce greater short‐term improvement in selected texture‐ and firmness‐related outcomes vs. the comparator formulation.

The discordance across endpoints is also informative. Group differences were more apparent in investigator‐ and participant‐rated texture and firmness than in the adapted full‐face WSRS or exploratory imaging measures. One possible explanation is that surface qualities such as smoothness and firmness may be more readily detected over a 12‐week interval than broader wrinkle severity changes. In addition, imaging‐based endpoints may have been insufficiently sensitive to modest short‐term changes in this small sample. The higher satisfaction reported in the REGF cohort is therefore notable, although it should be interpreted cautiously because the questionnaire used in this study was not validated.

In addition to objective and participant‐reported skin outcomes, this study incorporated an assessment of appearance‐related social perception using ratings from blinded lay evaluators who reviewed standardized photographs. Appearance‐based perception has been studied in surgical and minimally invasive aesthetic literature, but comparable work in topical cosmetic interventions remains limited [29, 30, 31]. Such research has reported changes in dating success (or “mating success” as is described in this sphere) and financial success, or rather “CEO success.” [29, 32, 33, 34] In the present study, lay‐evaluator ratings showed small and inconsistent differences across impression domains, and the instrument used was not validated for the constructs assessed. The observed reduction in perceived age in both groups is of interest, but the magnitude of change was modest and may fall within expected inter‐rater variability for photographic age estimation [35]. These findings therefore do not support strong conclusions regarding psychosocial benefit, but they do suggest that appearance‐related perception may be a reasonable exploratory outcome for future topical studies when measured with more rigorous instruments.

Several methodological considerations warrant emphasis. First, this was a small, single‐center pilot study with female‐only enrollment, which limits generalizability. As well, the 12‐week follow‐up period may be sufficient to capture changes related to epidermal renewal and surface texture, but it is relatively short for detecting slower dermal remodeling endpoints that depend on collagen and elastin turnover. The study was not powered for definitive between‐group comparisons across numerous exploratory endpoints, and some instruments used here were not validated. Finally, all participants used the same standardized adjunct cleanser and sunscreen regimen. While this approach reduced variability from uncontrolled baseline skincare routines, it introduces potential residual confounding (including brand‐associated expectancy effects or formulation synergy) that cannot be fully excluded. While this variable was not controlled, this practice aligns with comparative studies investigating topical EGF formulations and in broader biomedical research [6, 26, 27, 28, 36, 37, 38]. Larger, longer‐duration studies with more diverse populations, pre‐specified primary endpoints, and validated patient‐reported and perception‐based measures are needed to determine the robustness and clinical relevance of these preliminary signals.

## 5 Conclusion

This pilot randomized, double‐blinded study suggests that the peptide‐based REGF formulation may provide greater short‐term improvement in investigator‐ and participant‐rated skin texture and firmness than the conditioned‐media comparator over 12 weeks. Because wrinkle, imaging, and psychosocial perception endpoints showed limited or exploratory differences, these findings should be interpreted as hypothesis‐generating. Larger, longer‐duration studies with more diverse enrollment and standardized patient‐reported and perception‐based instruments are warranted.

## Author Contributions

Brittany Abud, contributed to supervision, project administration, writing – original draft, review and editing. Lander McGinn, contributed to formal analysis, validation, visualization, and writing – original draft, review and editing. Elvira Cawthon, contributed to formal analysis, writing – review and editing. Ashley Pankey, contributed to the visualization and writing of the original draft. Nimit Gandhi, contributed to data curation, project administration, supervision, and writing – review and editing. Catherine Carvajal, contributed to formal analysis, data curation, writing review and editing. Steven Dayan, contributed to project conceptualization, funding acquisition, investigation, methodology, resources, and writing review and editing.

## Funding

This work was supported by DRMTLGY.

## Ethics Statement

The study protocol received WCG institutional review board (IRB) approval for protocol 20 250 256.

## Consent

Written informed consent was obtained from all participants prior to participation, with explicit permission to publish photos.

## Conflicts of Interest

The authors declare no conflicts of interest.

## Supporting information


**Appendix S1:** 22‐item subject satisfaction questionnaire.

## Data Availability

The data that support the findings of this study are available on request from the corresponding author. The data are not publicly available due to privacy or ethical restrictions.
