# Supplementary material for: A Randomized, Double‐Blinded Pilot Study Comparing Synthetic Versus Human‐Derived Topical Epidermal Growth Factor for Facial Rejuvenation and Psychosocial Perception
Source: J Cosmet Dermatol. 2026 May 18;25:e70927. doi: 10.1111/jocd.70927 (PMC13184420; doi:10.1111/jocd.70927)
Supplement: Supplementary file 1 — Appendix S1: 22‐item subject satisfaction questionnaire. [file JOCD-25-e70927-s001.docx]

| ***Subject Satisfaction Questionnaire*** |
| --- |
| \|  \| **Yes** \| **No** \| \| --- \| --- \| --- \| \| “The test product reduces the appearance of crow’s feet around the eyes.” \|  \|  \| \| “The test product reduces the appearance of fine lines and wrinkles.” \|  \|  \| \| “The test product makes my skin feel firmer.” \|  \|  \| \| “The test product makes my skin look more plump.” \|  \|  \| \| “The test product makes my skin feel more elastic.” \|  \|  \| \| “The test product feels soothing to my skin.” \|  \|  \| \| “The test product did not cause the skin to look or feel irritated.” \|  \|  \| \| “The test product makes my skin appear brighter.” \|  \|  \| \| “The test product improved my skin’s lack luster/dull appearance.” \|  \|  \| \| “The test product makes my skin feel smoother.” \|  \|  \| \| “The test product improved my skin texture.” \|  \|  \| \| “The test product makes my pores less visible.” \|  \|  \| \| “The test product makes my skin appear more nourished/healthy.” \|  \|  \| \| “The test product made my skin appear more balanced.” \|  \|  \| \| “The test product made my skin appear less oily.” \|  \|  \| \| “The test product makes my skin feel more moisturized.” \|  \|  \| \| “The test product has a calming effect on my skin.” \|  \|  \| \| “The test product makes my face appear more youthful.” \|  \|  \| \| “The test product improves my skin’s clarity, providing a healthy, even tone.” \|  \|  \| \| “The test product was effective.” \|  \|  \| \| “The test product improves my skin’s overall appearance.” \|  \|  \| \| “Is it important to you that this product does not use any human byproduct, such as placenta?” \|  \|  \| |

Appendix 1. 22-item subject satisfaction questionnaire.
